# Evaluating efficacy, safety, and innovation in skin care applications of essential oils: a systematic review

**DOI:** 10.3389/fmed.2025.1589691

**Published:** 2025-08-21

**Authors:** Camila Pezantes-Orellana, Fátima German Bermúdez, José Montalvo, Tamara Packer, Andrea Orellana-Manzano

**Affiliations:** ^1^NovaVita, Guayaquil, Ecuador; ^2^Laboratorio para Investigaciones Biomédicas, Facultad de Ciencias de la Vida, Escuela Superior Politécnica del Litoral (ESPOL), Guayaquil, Ecuador; ^3^Licenciatura en Nutrición y Dietética, Facultad de Ciencias de la Vida, Escuela Superior Politécnica del Litoral, ESPOL, Guayaquil, Ecuador; ^4^Facultad de Ciencias de la Salud, Universidad Espíritu Santo, Samborondón, Ecuador

**Keywords:** essential oils, cosmetic product, bioactive compounds, skin conditions, dermocosmetic application

## Abstract

Essential oils have emerged as popular natural alternatives to synthetic ingredients in cosmetic products, drawing attention for their therapeutic potential in treating skin conditions like acne, psoriasis, and atopic dermatitis. This systematic review analyzed 70 studies from the past decade across multiple databases to evaluate their effectiveness and safety in derma cosmetic applications. The research encompassed clinical trials, *in vitro* studies, and *in vivo* experiments examining various essential oils in different cosmetic formulations, including lavender (*Lavandula angustifolia*), tea tree (*Melaleuca alternifolia*), chamomile (*Matricaria chamomilla*), peppermint (*Mentha piperita*), hemp (*Cannabis sativa*), *Euphorbia characias*, hierba de San Guillermo (*Agrimonia* *eupatoria*) and eucalyptus (*Eucalyptus globulus*). Key findings demonstrated that tea tree oil was particularly effective for acne treatment, while lavender and rosemary oils showed promising anti-inflammatory and anti-aging properties. Plant extracts from *Ocimum gratissimum* and *Matricaria chamomilla* also yielded positive results for skin health improvement. Various formulations such as gels, creams, and serums showed different levels of effectiveness in enhancing skin hydration, elasticity, and overall appearance. While the clinical evidence suggests significant potential for essential oils in cosmetic and dermatological applications, researchers emphasize the need for more comprehensive, longterm clinical trials to establish their efficacy and safety profiles fully.

## Graphical Abstract

The Selection process for studies on essential oils in cosmetic applications. 
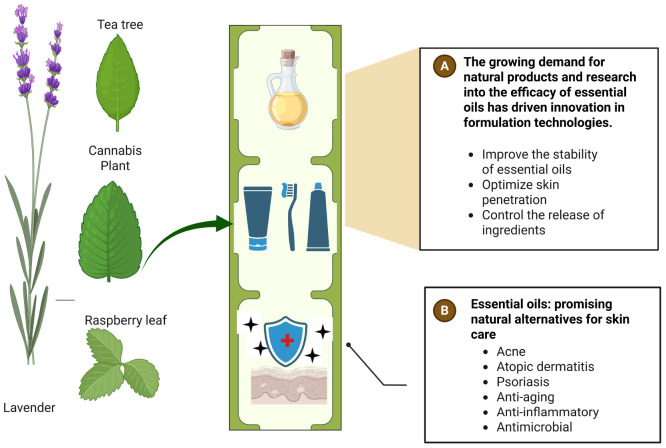

## 1 Introduction

Essential oils (EOs) have gained significant traction in contemporary cosmetic products, offering natural alternatives to synthetic components (1). EOs, derived from various plant parts through distillation (2) or cold pressing (1), have a wide range of bioactive compounds with potential skin benefits (3); these concentrated plant extracts are valued for their therapeutic properties and pleasant fragrances (3). Lavender (4), tea tree (5), and rosemary (6) oils are among the most popular in skincare formulations due to their antimicrobial, anti-inflammatory, and anti-age properties (7–10) for skin conditions. This affects millions worldwide, with varying prevalence between children and adults (11). In children, atopic dermatitis (eczema) is widespread, affecting up to 20% of children in developed countries (12). Adults commonly experience acne, rosacea, and psoriasis, with adult acne affecting up to 15% of women (13–15); environmental factors, genetics, hormonal changes, and lifestyle choices contribute significantly to these conditions (16). As a result, currently the cosmetic industry has significantly shifted toward natural and plant-based ingredients (17–19, 20) and The global essential oils market size in 2022 is expected to grow at a Compound Annual Growth Rate (CAGR) of 7.4% from 2023 to 2030 (21).

The cosmetic industry has significantly shifted toward natural and plant-based ingredients, with essential oils gaining prominence (16). This trend is driven by increasing consumer awareness of potential side effects from synthetic chemicals and a desire for “natural” beauty products (17, 18). Essential oils, derived from various plant parts through distillation (19) or cold pressing (1), offer a wide range of bioactive compounds with potential skin benefits (22). The global essential oils market size in 2022 is expected to grow at a Compound Annual Growth Rate (CAGR) of 7.4% from 2023 to 2030 (21). This growth is partly fueled by the incorporation of essential oils in cosmetic products (4). Lavender (5), tea tree (6), and rosemary (7) oils are among the most popular in skincare formulations due to their antimicrobial, anti-inflammatory, and anti-age properties (8–10).

The use of essential oils (EOs) in cosmetics presents challenges despite their general safety at low concentrations. Regulatory oversight remains minimal, as the Food and Drud Administration (FDA) does not require pre-approval for their use in fragrances and cosmetics, assuming compliance with labeling guidelines (23). Nevertheless, *in vitro* and *in vivo* studies support their efficacy, reinforcing consumer interest in natural skincare (24–27). To mitigate potential risks, the International Fragrance Association (IFRA) has established guidelines on allergenic components and permissible concentrations to enhance product safety (28).

EOs have been the subject of several scientific reviews in recent years, standing out for their therapeutic potential in the treatment of skin diseases. A systematic analysis of clinical trials showed that EOs, such as tea tree oil, have comparable or superior efficacy to conventional treatments such as acne and topical fungal infections, with a lower incidence of adverse side effects (29). In addition, a scoping review on the use of EOs in inflammatory skin conditions, such as dermatitis, psoriasis, and rosacea, indicated that these oils are well tolerated and offer an attractive alternative to traditional pharmacological treatments (30–32).

However, there are limitations to the available evidence. Although ECs show promise, the methodological quality of many studies is variable, with small sample sizes and lack of standardization in oil preparation. This may affect the consistency and generalizability of results. In addition, although EOs are generally well tolerated, adverse effects such as dermatitis have been reported, especially with oils such as lavender, peppermint, and tea tree, underscoring the need for caution in their use. Therefore, more rigorous research is required to establish protocols for safe and effective use of EOs in dermatology (30).

Essential oils have been widely recognized for their diverse bioactivities, including antioxidant, anti-inflammatory, antibacterial, antifungal, antiviral, skin-barrier repairing, and anticancer properties. These concentrated plant extracts are rich in bioactive phytochemicals and have been used for centuries in traditional medicine to address various dermatological issues such as acne, inflammation, aging, and dryness (29). These metabolites, secondary principal plant compounds, include terpenes, phenylpropanoids, and other aromatic compounds (32, 33). Terpenes, such as limonene in citrus oils and α-pinene in rosemary oil, exhibit antimicrobial and anti-inflammatory properties. These metabolites interact with skin cells (34) and the microbiome in complex ways, influencing cellular processes and microbial populations (35). These natural compounds, extracted from various plant sources, have gained significant attention in dermatological applications due to their multifaceted therapeutic potential and relatively favorable safety profiles compared to synthetic alternatives (36, 37).

For instance, EOs such as lavender (*Lavandula angustifolia*), tea tree (*Melaleuca alternifolia*), chamomile (*Matricaria chamomilla*), peppermint (*Mentha piperita*), hemp (*Cannabis sativa*), *Euphorbia characias*, hierba de San Guillermo (*Agrimonia eupatoria*) and eucalyptus (*Eucalyptus globulus*) are widely utilized in dermocosmetics for their multifaceted therapeutic properties. Lavender oil is prized for its anti-inflammatory and antimicrobial effects, making it effective in treating acne and soothing irritated skin, while also promoting skin barrier repair (38, 39). Tea tree oil, rich in terpenes like terpinen-4-ol, is a potent antibacterial and antifungal agent, commonly used in formulations targeting acne and scalp conditions (40–42). Chamomile oil, with its high bisabolol content, reduces redness and inflammation, making it ideal for sensitive or eczema-prone skin (42, 43). Peppermint and eucalyptus oils provide cooling sensations and antimicrobial benefits, often incorporated into products for oily or congested skin due to their ability to regulate sebum production and combat microbial overgrowth (43–46). *Agrimonia eupatoria*, *Bursera morelensis*, and *Jatropha neopauciflora* are noted for their wound-healing capabilities, promoting tissue repair and regeneration (47). In addressing photoprotection and hyperpigmentation, oils such as *Astragalus gombiformis* and *Euphorbia characias* exhibit strong UV-protective properties, acting as natural sunscreen agents (48, 49). For skin brightening, oils like *Aerva lanata* and *Schisandra chinensis* demonstrate potent anti-tyrosinase activity, reducing melanin synthesis to combat uneven skin tone (50, 51).

Integrating essential oils into cosmetic products has driven innovation in formulation technologies (52). Microencapsulation has emerged as a key technology, allowing for the controlled release of essential oil components and improved stability (52–55). This technique involves encasing tiny droplets of essential oils within a protective shell, often made of polymers or lipids (56). Nanoemulsion technology is another advancement, creating ultra-small droplets of essential oils dispersed in water, enhancing their penetration into the skin and improving efficacy (57). Liposomal delivery systems are also being employed, where essential oil components are encapsulated within lipid vesicles, mimicking the structure of cell membranes for better absorption (53). These technologies enhance the stability and efficacy of essential oils and allow for precise dosing, reducing the risk of skin irritation. Furthermore, green extraction methods, such as supercritical CO2 extraction, are increasingly used to obtain essential oils, ensuring higher purity and reducing environmental impact (58). The cosmetic industry is also exploring synergistic blends of essential oils, combining different oils to enhance therapeutic effects while minimizing potential adverse reactions.

The growing preference for essential oil-based cosmetics over traditional pharmaceutical formulations stems from several factors. Firstly, essential oils offer a more holistic approach to skincare, addressing specific skin conditions and promoting overall skin health and well-being (59). Unlike many pharmaceutical products that target single pathways or symptoms, essential oils can provide multiple benefits due to their complex composition (22, 60). Secondly, essential oils often have fewer side effects than synthetic pharmaceutical ingredients, making them suitable for long-term use in daily skincare routines (61). As research in this field progresses, the integration of essential oils in evidence-based skincare will likely increase, bridging the gap between traditional natural remedies and modern dermatological science.

Recent clinical research has increasingly supported the efficacy of tea tree oil (TTO) products in the management of acne vulgaris, with several studies in the past 5 years demonstrating promising results. A notable randomized clinical trial compared a novel tea tree oil nanoemulsion containing adapalene gel (TTO NE + ADA Gel) to a standard 0.1% adapalene marketed gel in 100 patients over 12 weeks. The study found that the TTO NE + ADA Gel led to a significantly greater reduction in total, inflammatory, and non-inflammatory acne lesions at all measured intervals (weeks 4, 8, and 12), as well as a more pronounced decrease in the acne severity index (*p* < 0.001 for all comparisons). Importantly, while dryness was the most common adverse effect in both groups, the overall safety profile of the TTO NE + ADA Gel was comparable to the conventional adapalene gel, suggesting that the addition of tea tree oil may enhance efficacy without increasing significant side effects (1).

Another recent investigation evaluated the use of tea tree oil gel and face wash in patients with mild to moderate acne over 12 weeks. Participants applied the tea tree oil products twice daily, significantly reducing mean total lesion counts from 23.7 at baseline to 10.7 at 12 weeks (*P* < 0.0001). The investigator global assessment (IGA) scores improved significantly during the study. No serious adverse events were reported, and minor local effects such as peeling, dryness, and scaling resolved without intervention. The study concluded that tea tree oil products are effective and well tolerated for mild to moderate acne, supporting their use as a viable alternative or adjunct to conventional therapies (2). These findings underscore the growing evidence for tea tree oil as an effective and safe option for acne management, meriting further comparative trials against standard patent medicines and another study comparing TTO to a novel fermented extract found that while both reduced inflammatory lesions, the new compound acted faster and had a better safety profile (3).

The integration of essential oils into advanced delivery systems is further supported by comprehensive reviews that explore their components and mechanisms of action in cosmetic formulations. Guzmán and Lucia analyze the bioactive constituents of EOs, such as terpenes and phenolic compounds, which contribute to their multifaceted roles, including antioxidative, antimicrobial, and anti-aging effects (11). Dontje et al. (62) expand on this by reviewing the therapeutic potential of EOs in managing inflammatory skin conditions, highlighting that nanoformulations can significantly enhance the efficacy of EOs by improving skin targeting and reducing adverse reactions. This synergy between EO bioactivity and nanotechnology is crucial for developing the next generation of dermocosmetics that offer both safety and enhanced performance (12). Bongunuri et al. provide a comprehensive review of the antioxidant properties of essential oils (EOs) and the recent advancements in their incorporation into nanoformulations for cosmetic applications. The authors emphasize that essential oils possess potent free radical scavenging abilities due to their rich content of phenolic and terpenoid compounds, which help protect skin cells from oxidative stress and premature aging. However, the volatile and hydrophobic nature of EOs limits their direct use in skincare products. To overcome these challenges, the review highlights the development of nanoencapsulation techniques such as nanoemulsions, liposomes, and solid lipid nanoparticles, which enhance stability, bioavailability, and controlled release of EOs. These nano formulations improve skin penetration, reduce irritation, and increase the shelf-life of cosmetic products, making them highly promising for modern dermocosmetic innovations (13).

Similarly, Javed et al. focus on the role of essential oils as dermocosmetic agents, detailing their mechanisms of action and the benefits of nanolipidic delivery systems for maximizing skincare efficacy (14) the article discusses how EOs exert antimicrobial, anti-inflammatory, and antioxidant effects that contribute to skin health and the treatment of various dermatological conditions. The authors underscore that nanolipidic carriers, including solid lipid nanoparticles and nanostructured lipid carriers, significantly enhance the solubility, stability, and skin permeability of EOs. This leads to improved therapeutic outcomes such as enhanced hydration, reduced inflammation, and protection against environmental damage. The review also points out that these advanced delivery systems can mitigate common side effects of EOs, such as skin irritation, thereby broadening their applicability in safe and effective cosmetic formulations (63).

Looking toward future perspectives, the prospects of EO-loaded nanosystems in skincare are promising, with ongoing research focusing on optimizing formulation strategies to achieve multifunctional benefits. Kashyap et al. and Achagar et al. provide insights into designing nano-emulsions, liposomes, and solid lipid nanoparticles as carriers that improve EO stability and skin compatibility (15). Moreover, Yihan et al. discuss recent advances in planting EO drug delivery systems, emphasizing their potential in photoprotection, wound healing, and anti-aging therapies. These developments reflect a growing trend toward integrating traditional botanical ingredients with cutting-edge nanotechnology to create innovative, effective, and consumer-friendly skincare products (16).

## 2 Materials and methods

This systematic review follows the PRISMA (Preferred Reporting Items for Systematic Reviews and Meta-Analysis) (64) verification protocol with keywords in the search strategy as Essential oils, cosmetic products, Bioactive Compounds, Skin Conditions, previously published Clinical case Trials in human participants were used along with the use of search operators AND, OR. The study focuses on using essential oils in cosmetic products, particularly their anti-inflammatory, antimicrobial, and anti-aging effects when applied topically. The research examines various essential oils such as tea tree oil, lavender, and rosemary, and plant extracts like *Ocimum gratissimum* and *Matricaria chamomilla* for their potential benefits in addressing skin conditions including acne, psoriasis, and atopic dermatitis, as well as general anti-aging effects. The search for articles was conducted in humans, specifically in PubMed and the Clinical Research database.

The study selection process was carried out in two stages. In the first, the titles and abstracts of the studies were reviewed based on the inclusion and exclusion criteria. They identified studies that met the inclusion criteria and excluded those that did not. In the second stage, they reviewed full articles and excluded studies that did not meet the inclusion criteria. Customized search strategies were developed for the PubMed and Clinical Research bibliographic databases. The search was performed directly and included all articles published in the last 10 years without language restrictions.

The following data were recorded for the studies included in the Pubmed database: author(s), article title, year of publication, type of essential oil, type of cosmetic product, bioactive compound, functional characteristics, and main results. For the studies incorporated in the Clinical Research database, the following data were recorded: NCT Number, Study Title, Study URL, Study Status, Conditions, Interventions, Primary Outcome Measures, Secondary Outcome Measures, Other Outcome Measures, Sponsor Collaborators, Sex, Age, Phases, Enrollment, Study Type, Study Design, Results First Posted and Study Documents. Finally, information is provided on the design of the studies, the countries where they were conducted, the size of the population studied, the types of essential oils used, the cosmetic formulations employed, and the bioactive compounds present in each oil. In addition, DOI identifiers are included.

### 2.1 Eligibility criteria

#### 2.1.1 Inclusion and exclusion criteria

Studies were considered eligible if they met the following criteria: (1) clinical studies in human subjects, (2) Studies investigating the health benefits of essential oils in cosmetic applications, (3) studies without any language restrictions, (4) articles published in the last 10 years and (5) manuscripts in journals with an impact index.

The following studies were excluded: (1) not meeting the objectives of investigating essential oils in cosmetics, (2) cell lines, mouse models, or another experimental, (3) not meeting the search criteria, (4) book chapters or books, (5) Reviews and Meta-analysis, (6) articles published more than 10 years ago, and (7) Studies not found in the specified databases (PubMed and Clinical Research).

#### 2.1.2 Search strategies

To ensure a comprehensive review, a detailed search strategy was implemented across PubMed and Clinical Research databases. The search used keywords and phrases connected by Boolean operators (AND, OR) to capture relevant studies. The primary search string was constructed as follows: ((“Essential oils” OR “Volatile oils” OR “Aromatherapy oils” OR “Plant oils” OR “Plant extracts”) AND (“Cosmetic products” OR “Skincare” OR “Topical application” OR “Dermal absorption”) AND (“Skin conditions” OR “Acne” OR “Psoriasis” OR “Atopic dermatitis” OR “Anti-aging”) AND (“Bioactive compounds” OR “Active ingredients” OR “Phytochemicals”)) AND (“Clinical trials” OR “*In vivo* studies” OR “Human studies”). Specific essential oils were also included in the search: (“Tea tree oil” OR “Lavender oil” OR “Rosemary oil” OR “Cinnamon oil” OR “Cumin oil” OR “Coriander oil” OR “Parsley oil”) were included to capture broader aspects of essential oil research.

## 3 Results

### 3.1 Study selection

**FIGURE 1:**
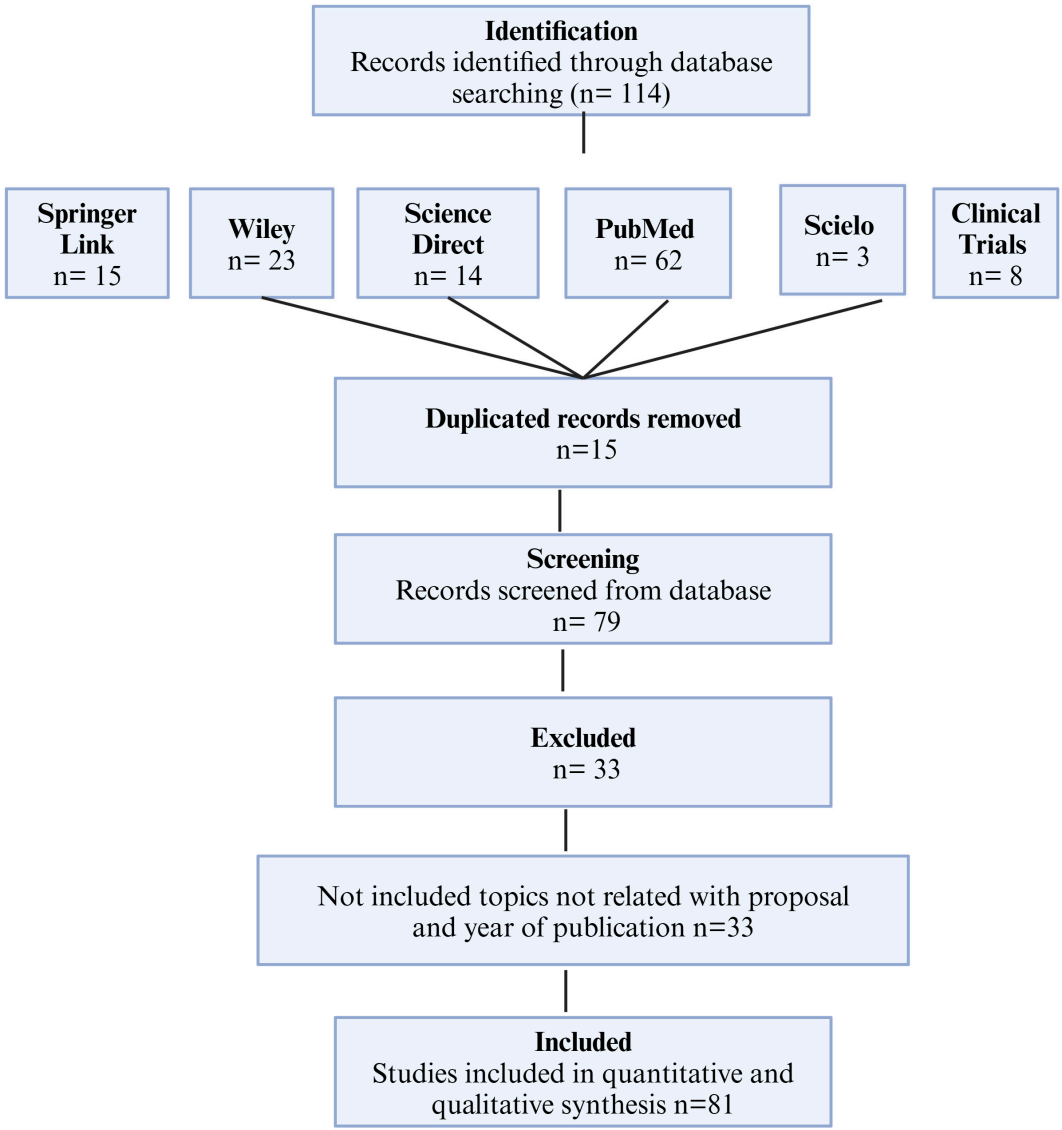
Study design of the selection process for studies on essential oils in cosmetic applications.

All the authors searched using the inclusion and exclusion criteria previously described in the databases, including the Cochrane Library. Subsequently, one author counted all the references and identified 133 citations in the PubMed Clinical Trials electronic database and Cochrane Library. After thoroughly reviewing the abstracts in the second phase, another author excluded 33 articles with 15 duplicates. Finally, we identified five additional articles through PubMed and reviewed their full text. Thus, this study included 80 references (Figure 1).

### 3.2 Study characteristics

The studies analyzed in this research were conducted in various countries, including Italy, Australia, and Nigeria, and published between 2016 and 2023 in English and Spanish. All selected studies focused on the use of essential oils in cosmetic products. The research primarily investigated the properties of these oils, such as their anti-inflammatory, antimicrobial, and anti-aging effects when applied topically. The studies have produced significant findings on the benefits of using essential oils in cosmetic formulations, including improvements in skin conditions like acne, psoriasis, and atopic dermatitis, as well as anti-aging effects.

Table 1 outlines studies on essential oils in cosmetic applications, highlighting the types of products, essential oils used, and main bioactive compounds identified. It covers research from various countries, focusing on skin conditions like acne, atopic dermatitis, and psoriasis. The table showcases diverse formulations, including gels, creams, and lotions, demonstrating the versatility of essential oils in skincare. Notable findings include the efficacy of tea tree oil for acne treatment and the anti-inflammatory properties of various plant extracts. Table 2 outlines clinical trials investigating essential oils and plant extracts in cosmetic and dermatological applications between 2016 and 2024. It covers a range of interventional and randomized studies exploring stress relief, skin hydration, acne treatment, pain management, and hair loss prevention. Various essential oils, including lavender, tea tree, and CBD, were studied in different formulations. The trials employed diverse outcome measures, from physiological responses to clinical assessments, highlighting the growing interest in essential oils for cosmetic and dermatological uses.

**TABLE 1:** Clinical efficacy of essential oils and botanical extracts in topical formulations for acne, aging, and inflammatory skin conditions.

	Article title	Essential oil	Type of cosmetic product	Bioactive compound	Functional characteristic	Main results	DOI
1	Anti-aging and brightening effects of a topical treatment containing vitamin C, vitamin E, and raspberry leaf cell culture extract: A split-face, randomized controlled trial	raspberry leaf extract (*Rubus idaeus*)	Topical cream	Vitamin C, Vitamin E, Polyphenols	Anti-aging, Brightening	Significant improvement in skin radiance, texture and fine wrinkles	doi: 10.1111/jocd.13305
2	Tea tree oil gel for mild to moderate acne; a 12 week uncontrolled, open-label phase II pilot study	Tea Tree Oil (*Melaleuca alternifolia*)	Gel	Terpinen-4-ol	Antibacterial, Anti-acne	The use of tea tree oil products significantly improved mild to moderate acne and the products were well tolerated.	doi: 10.1111/ajd.12465
3	Essential Oil of Matricaria chamomilla Alleviate Psoriatic-Like Skin Inflammation by Inhibiting PI3K/Akt/mTOR and p38MAPK Signaling Pathway	German Chamomile Essential Oil (MCEO)	Essential oil	Unspecified	Anti-inflammatory	MCEO acts by inhibiting PI3K/Akt/mTOR and p38MAPK signaling pathways, which are involved in the inflammation and pathogenesis of psoriasis.	doi: 10.2147/CCID.S445008
4	The efficacy of 5% topical tea tree oil gel in mild to moderate acne vulgaris: a randomized, double-blind placebo-controlled study	Tea Tree Oil (Melaleuca alternifolia)	Gel	Unspecified	Anti-inflammatory, Antibacterial Promotes reduction of acne lesions	Was shown to be 3.55 times more effective than placebo in reducing total acne lesion count (TLC). It was 5.75 times more effective in improving the acne severity index (ASI). Similar and tolerable side effects in both groups (treatment and placebo)	doi: 10.4103/0378-6323.30646
5	Treatment of acne with a combination of propolis, tea tree oil, and Aloe vera compared to erythromycin cream: two double-blind investigations	Tea tree oil, Propolis, Aloe vera	Topical cream	Propolis: flavonoids; Tea tree oil: terpenes; Aloe vera: polysaccharides	Antimicrobial and anti-inflammatory	Reduction of acne lesions between 23.7% and 62.1% after 4-8 weeks of use. Effective in combination with other extracts.	doi: 10.2147/CPAA.S180474

**TABLE 2:** Clinical trials of cosmetic products with natural ingredients: a comparative analysis of efficacy, safety and user perception in various dermatological conditions.

NCT number	Study Title	Study URL	Study Status	Conditions	Interventions	Primary outcome measures	Secondary outcome measures	Other outcome measures	Sponsor
**NCT04127279**	Stress Relief Properties of a Cosmetic Routine	https://clinicaltrials.gov/study/NCT04127279	COMPLETED	Stress Physiology |Stress, Psychological	BIOLOGICAL: Cream	• Acute autonomic stress responsivity (heart rate via ECG) • HPA axis stress responsivity (salivary cortisol levels) • High frequency component power of RR interval spectrum. Assessment timeline Day 0 (before/after cream application), Day 29.	• Perceived Stress Scale (PSS) questionnaire (0-40 scale) • Profile of Mood States (POMS) questionnaire • Behavioral coping style assessment	Unspecified	University of Parma
**NCT04045119**	Effect of a Facial Cream Containing Cannabidiol and Hemp Oil on Skin Hydration and Acne-prone Skin	https://clinicaltrials.gov/study/NCT04045119	COMPLETED	Cosmetic Acne	OTHER: Topical Moisturizer	• Skin hydration evaluation (single application) • Water content measurement via capacitance • Hydration effect of periodic application. Assessment timeline 1 hour, 3 hours, 2 weeks, 4 weeks	• Instrumentally assessed oily skin measurements • Skin biochromophores evaluation • Subjective oily skin assessment (0–10 VAS) • Quality of life (OSSIQ questionnaire)	NA	Avicanna Inc
**NCT04045314**	Effect of an Emollient Cream Containing 0.5% Cannabidiol and 1% Hemp Oil in the Hydration and Erythema of the Skin	https://clinicaltrials.gov/study/NCT04045314	COMPLETED	Cosmetics; Eczema	OTHER: Topical moisturizer	• Hydration effect on facial skin and volar forearm • Electrical properties measurement as water content indicator. Assessment timeline 1 hour, 3 hours, 2 weeks, 4 weeks	• Short/long-term erythema assessment • Viscoelastic skin properties • Trans-epidermal water loss measurement • Evaluator-assessed irritation scoring	NA	Avicanna Inc
**NCT05202366**	An Open-label Study Evaluating the Effectiveness of CGB-400 Topical Gel for Fungal Infection	https://clinicaltrials.gov/study/NCT05202366	UNKNOWN	Fungal Infection |Onychomycosis |Tinea Unguium	DRUG: CGB-400 Topical Gel	• Investigator Global Assessment (IGA) of affected toenails Severity scale: Clear (0%) to Severe (>50–75% involvement). Assessment timeline Investigator Global Assessment (IGA) of affected toenails Severity scale: Clear (0%) to Severe (>50–75% involvement)	• Percent clear area on affected toenails • Visual inspection with photography	The Subject Global Assessment consists of a questionnaire at baseline describing the severity of fungal growth, its causes, attempts at management, and earlier treatments used. The participants may also describe any other information that they feel is relevant to their experience during the 24-week study period. The Investigator (or designate) will ask the following questions at the Baseline visit and subject’s answers will be recorded: 1. How frequently do you typically experience fungal growth each month? 2. How do you rate your fungal growth today relative to other times?	CAGE Bio Inc.
								3. Are you aware of specific triggers that aggravate fungal growth? 4. How do you deal with or treat fungal growth? 5. What other products have you tried in the past for managing/treating fungal growth? 6. Are there any other aspects of fungal growth or experience that we should know about?, 24 weeks	
**NCT01657110**	Pilot Study to Evaluate Tea Tree Oil Gel for Facial Acne	https://clinicaltrials.gov/study/NCT01657110?intr=Tea%20tree%20oil%20gel&rank=1	COMPLETED	Cosmetic, Acne	Other: Tea tree oil	• Lesion count (inflamed and non-inflamed facial lesions) Investigator Global Assessment (5-point severity scale 0–4)	Decreased non-inflammatory lesion count Decrease in numbers of non-inflammatory lesions from baseline 12 weeks Decreased inflammatory lesion count Decrease in inflammatory lesion count from baseline 12 weeks Decrease in perceived facial oiliness Decrease in perceived facial oiliness from baseline 12 weeks	Mean tolerability score Mean tolerability will be determined as the average of the following; 12 weeks Erythema Scaling Peeling Burning Induration Dryness These six parameters will be measured using a 5-point scale (0: None, 1: Minimal, 2: Mild, 3: Moderate, 4: Severe). The study assesses mean tolerability at 12 weeks by averaging the six skin reactions scored 0 to 4 and also records the incidence, nature, and severity of any local or systemic adverse events. Mean tolerability will be the average of these scores. The frequency of adverse events Any local or systemic adverse events will be recorded including the type of reaction and severity (on a 5-point severity scale where 0 = none and 4 = severe). 12 weeks	The University of Western Australia
**NCT04769271**	Intra-pocket Application of Tea Tree Oil Gel in the Treatment of Stage-2 Periodontitis	https://clinicaltrials.gov/study/NCT04769271?intr=Tea%20tree%20oil%20gel&rank=3	COMPLETED	Cosmetic, Periodontitis	Procedure: Scaling and Root Planing Drug: Scaling and Root Planing with tea tree oil	• Probing depth measurement • Clinical attachment loss assessment • Bleeding on probing evaluation • Biochemical assessment (MMP-8 levels). Assessment timeline Up to 6 months	NA	NA	Alexandria University
**NCT06060834**	Impact of a Topical Cosmetic Product on Women’s Hair	https://clinicaltrials.gov/study/NCT06060834?intr=%20lemon%20%20oil%20cosmetic%20&rank=1	Active, not recruiting	Cosmetic, Hair Thinning, Hair Loss	Dietary Supplement: Topical Botanical Agent	• Hair density via “MyHairCounts” application • 60-second hair count (hairs lost during combing) • Self-assessment of well-being (0–10 scale). Assessment timeline Baseline, 2 weeks, 1 month	• Vital signs monitoring (BP, HR) • Product assessment (smell, ease of use) • Hair self-assessment (thickness, fullness, etc.)	Change HistoryNo changes postedPrimary (Current) *ICMJE(Submitted: 2023-09-22) Hair Density [Time Frame: baseline] Hair density as determined by “MyHairCounts” applicationHair Density [Time Frame: After 2 weeks of treatment] Hair density as determined by “MyHairCounts” application Hair Density [Time Frame: after 1 month of treatment] It measures hair density, 60-second hair shedding, and self-report scales of well-being, product quality, and characteristics of hair at three time points-baseline, 2 weeks, and 1 month. Objective and subjective responses to treatment are measured through applications and visual analog scales. Hair density as determined by “MyHairCounts” application 60 second hair count [Time Frame: baseline] Number of hairs lost when hair is combed for 60 seconds 60 second hair count [Time Frame: After 2 weeks of treatment] Number of hairs lost when hair is combed for 60 seconds 60 second hair count [Time Frame: after 1 month of treatment] Number of hairs lost when hair is combed for 60 seconds Self-assessment of well-being [Time Frame: baseline] An adaptive visual analog scale will be used to self-assess feelings of confidence, self-consciousness, motivation, and attractiveness/beautifulness on a continuous scale from 0 (not at all) to 10 (extreme).	
								Self-assessment of well-being [Time Frame: After 2 weeks of treatment] An adaptive visual analog scale will be used to self-assess feelings of confidence, self-consciousness, motivation, and attractiveness/beautifulness on a continuous scale from 0 (not at all) to 10 (extreme). Self-assessment of well-being [Time Frame: After 1 month treatment]An adaptive visual analog scale will be used to self-assess feelings of confidence, self-consciousness, motivation, and attractiveness/beautifulness on a continuous scale from 0 (not at all) to 10 (extreme). Product assessment [Time Frame: after 1 month of treatment] An adaptive visual analog scale will be used to self-assess feelings on the smell, ease of use, and self-reported feeling of hair quality using a continuous scale from 1 (not at all) to 10 (Very much). Self-assessment of hair [Time Frame: baseline] An adaptive visual analog scale will be used to self-assess feelings on subject’s hair (thickness, fullness, shininess, texture, strength, appearance,	
								satisfaction using a continuous scale from 1 (extremely poor) to 10 (Excellent) as well as compliments, split ends on a scale from 1 (Never) to 10 (Very often), ease of styling 1 (very difficult) to 10 (very easy), and speed of hair growth 1 (slow) to 10 (very fast). Self-assessment of hair [Time Frame: After 2 weeks of treatment] An adaptive visual analog scale will be used to self-assess feelings on subject’s hair (thickness, fullness, shininess, texture, strength, appearance, satisfaction using a continuous scale from 1 (extremely poor) to 10 (Excellent) as well as compliments, split ends on a scale from 1 (Never) to 10 (Very often), ease of styling 1 (very difficult) to 10 (very easy), and speed of hair growth 1 (slow) to 10 (very fast). Self-assessment of hair [Time Frame: After 1 month of treatment]An adaptive visual analog scale will be used to self-assess feelings on subject’s hair (thickness, fullness,	
								shininess, texture, strength, appearance, satisfaction using a continuous scale from 1 (extremely poor) to 10 (Excellent) as well as compliments, split ends on a scale from 1 (Never) to 10 (Very often), ease of styling 1 (very difficult) to 10 (very easy), and speed of hair growth 1 (slow) to 10 (very fast).	
**NCT03016221**	Local Changes of Skin Characteristics After an Application of a Topical Product With a Warming or Cooling Effect (SRPT)	https://clinicaltrials.gov/study/NCT03016221?intr=rosemary%20oil%20gel&rank=2	COMPLETED	Cosmetic, Healthy, gel	Other: Axanova hot gelOther: Axanova activ gelOther: Perskindol Dolo GelShow 5 more interventions/treatments	• Local skin perfusion (laser speckle instrument) • Local skin redness (chromameter) • Skin surface temperature • Local muscle tissue oxygenation • Subjective temperature sensation. Assessment timeline 1 hour post-application	• Surrounding area measurements • Adjacent skin response evaluation	NA	University of Applied Sciences and Arts of Southern Switzerland
**Collaborators**		**Sex**	**Age**	**Phases**	**Enrollment**	**Study Type**	**Study Design**	**Results First Posted**	**Study Documents**
Unspecified		FEMALE	ADULT	PHASE4	40	INTERVENTIONAL	Allocation: RANDOMIZED |Intervention Model: PARALLEL |Masking: SINGLE (PARTICIPANT) |Primary Purpose: TREATMENT	NA	NA
Unspecified		ALL	ADULT, OLDER_ADULT	NA	54	INTERVENTIONAL	Allocation: NA |Intervention Model: SINGLE_GROUP |Masking: NONE |Primary Purpose: OTHER	NA	NA
Unspecified		ALL	ADULT, OLDER_ADULT	NA	49	INTERVENTIONAL	Allocation: NA |Intervention Model: SINGLE_GROUP |Masking: NONE |Primary Purpose: OTHER	NA	NA
John Peter Smith Hospital		ALL	ADULT, OLDER_ADULT	NA	15	OBSERVATIONAL	Observational Model: |Time Perspective	NA	NA
Royal Perth Hospital Hollywood Private Hospital Principal Investigator: Prasad Kumarasinghe, Royal Perth Hospital		ALL	16–45 years (Child, Adult)	NA	18	INTERVENTIONAL	Primary Purpose: Treatment Allocation: N/AInterventional Model: Single Group Assignment Masking: None (Open Label)	NA	NA
Alexandria UniversityStudy Chair: Maha R. Taalab, PhD, Faculty of Dentistry, Alexandria University, EgyptPrincipal Investigator: Dania M. Abdel Aziz, BDS, Faculty of Dentistry, Alexandria University, EgyptStudy Director: Sabah A Mahmoud, PhD, Medical Biochemistry and Molecular Biology department. Faculty of Dentistry, Alexandria University, Alexandria, Egypt. Study Director: Riham M El Moslemany, PhD, Pharmaceutics department. Faculty of Pharmacy, Alexandria University; Alexandria, Egypt.		ALL	20–40 years (Adult)	Phase 2	30	INTERVENTIONAL	Primary Purpose: Treatment Allocation: Randomized Interventional Model: Parallel Assignment Masking: Single (Outcomes Assessor)	https://pubmed.ncbi.nlm.nih.gov/33952216/	NA
University of Memphis Principal Investigator: Richard Bloomer, PhD, University of Memphis		FEMALE	35–70 years (Adult, Older Adult)	NA	15	INTERVENTIONAL	Primary Purpose: Treatment Allocation: N/A Interventional Model: Single Group Assignment Interventional Model Description: pre-post experimentMasking: None (Open Label)	NA	NA
University of Applied Sciences and Arts of Southern Switzerland		ALL	18–40 years (Adult)	NA	90	INTERVENTIONAL	Primary Purpose: Basic Science Allocation: Randomized Interventional Model: Parallel Assignment Masking: Triple (Participant Investigator Outcomes Assessor)	NA	NA

The results of this study (10) showed that the serum containing vitamin C, vitamin E, and raspberry leaf cell culture extract significantly improved skin color, elasticity, and radiance. There was also a marked improvement in skin smoothness, scaliness, and wrinkling. Adverse reactions were mild, including tingling sensations and tightness. In this 12-week study evaluating the efficacy of a tea tree oil gel for the treatment of mild to moderate facial acne, significant results were found: the mean facial lesion count decreased from 23.7 at baseline to 10.7 at the end of the study, and the investigator global assessment (IGA) score also showed significant improvement (8). The results showed anti-inflammatory activity: the cream containing propolis, tea tree oil, and Aloe vera was more effective in reducing the acne severity index (ASI) and total number of lesions (TLC) compared to 3% erythromycin cream. After 30 days of treatment, ASI was reduced by 66.7% in the PTAC group, while in the ERC group, the reduction was 49.7%. Likewise, TLC decreased by 63.7% in the PTAC group versus 46.5% in the ERC group, indicating a significantly higher efficacy of the natural ingredient-based treatment (CPAA) (65). The study showed a significant difference between tea tree oil gel and placebo in improving total acne lesion count (TLC) and acne severity index (ASI).

Regarding TLC and ASI, tea tree oil gel was 3.55 times and 5.75 times more effective than placebo, respectively. Side effects in both groups were relatively similar and tolerable (66). The results indicate that *Matricaria chamomilla* essential oil (MCEO) significantly reduced the elevation of inflammation in HaCaT keratinocyte cells stimulated by interleukin-22, tumor necrosis factor α, and lipopolysaccharide, which was related to the decreased activation of MCEO-mediated PI3K/Akt/mTOR and p38MAPK pathways. In this regard, MCEO contributed significantly to treating inflammatory skin diseases, as assessed by the PASI score, and HE is staining and inflammatory cytokine levels (9).

Table 2 summarizes clinical trials investigating essential oils and plant extracts in cosmetic and dermatological applications conducted between 2016 and 2024. These studies, primarily interventional and randomized, explore diverse applications, including stress relief, skin hydration, acne treatment, pain management, and hair loss prevention. Various essential oils such as lavender, tea tree oil, cannabidiol (CBD), and hemp oil were studied and incorporated into different formulations like creams, gels, and topical applications. The trials employed various outcome measures, from physiological responses (e.g., heart rate, skin hydration) to clinical assessments (e.g., acne lesion count, hair density) and subjective evaluations. Participants included males and females, typically adults and older adults, with study durations ranging from acute effects to 24 weeks. This overview highlights the growing interest in essential oils for cosmetic and dermatological uses, demonstrating their potential benefits across various skin and hair conditions and their effects on stress and well-being.

The study investigated the effect of an extract of *Myrothamnus flabellifolia* on stress reduction during the application of a cosmetic product as a potential to improve the psychological well-being of consumers by reducing the stress associated with using cosmetic products. It was observed that, after application of the active compound, cortisol levels in saliva decreased significantly. In contrast, levels of positive affect increased. Negative affect decreased, and there was a trend toward an increase in alpha wave activity in the brain, suggesting a state of relaxation (67).

The aim of the study (26) was to evaluate the short- and long-term moisturizing effect of a topical preparation containing CBD, rosemary extract, and hemp oil on facial skin to objectively measure changes in parameters related to skin water content, sebum production, and appearance, as well as the impact on the quality of life of participants with acne-prone skin. This includes instrumental and subjective measurements and standardized photography for blemish assessment. Similarly, this study (68) investigated the short- and long-term moisturizing effect of a topical preparation containing CBD and hemp oil on volar forearm skin and its impact on instrumentally measured erythema. It seeks to determine how the application of this product can improve skin hydration and reduce redness. On the other hand, the objective of this trial was to assess the effectiveness of CGB-400 topical gel for fungal infections and to evaluate the safety of the gel and its side effects (69).

Considering what has been presented above, these studies aimed to investigate the effectiveness of a tea tree oil gel. On the one hand, this trial (70) evaluated whether using the gel for 12 weeks improves acne severity in participants by counting lesions and assigning an overall acne severity score at different intervals (4, 8, and 12 weeks). In addition, we sought to provide evidence of the efficacy of this commercial product for the treatment of acne; we changed this trial (71) to the intra-pocket application of tea tree oil gel in the treatment of stage 2 periodontitis to evaluate the efficacy and safety of this treatment compared to conventional methods. We sought to determine whether tea tree oil gel can improve clinical outcomes, reduce inflammation, and promote periodontal health in patients with periodontitis.

**FIGURE 2:**
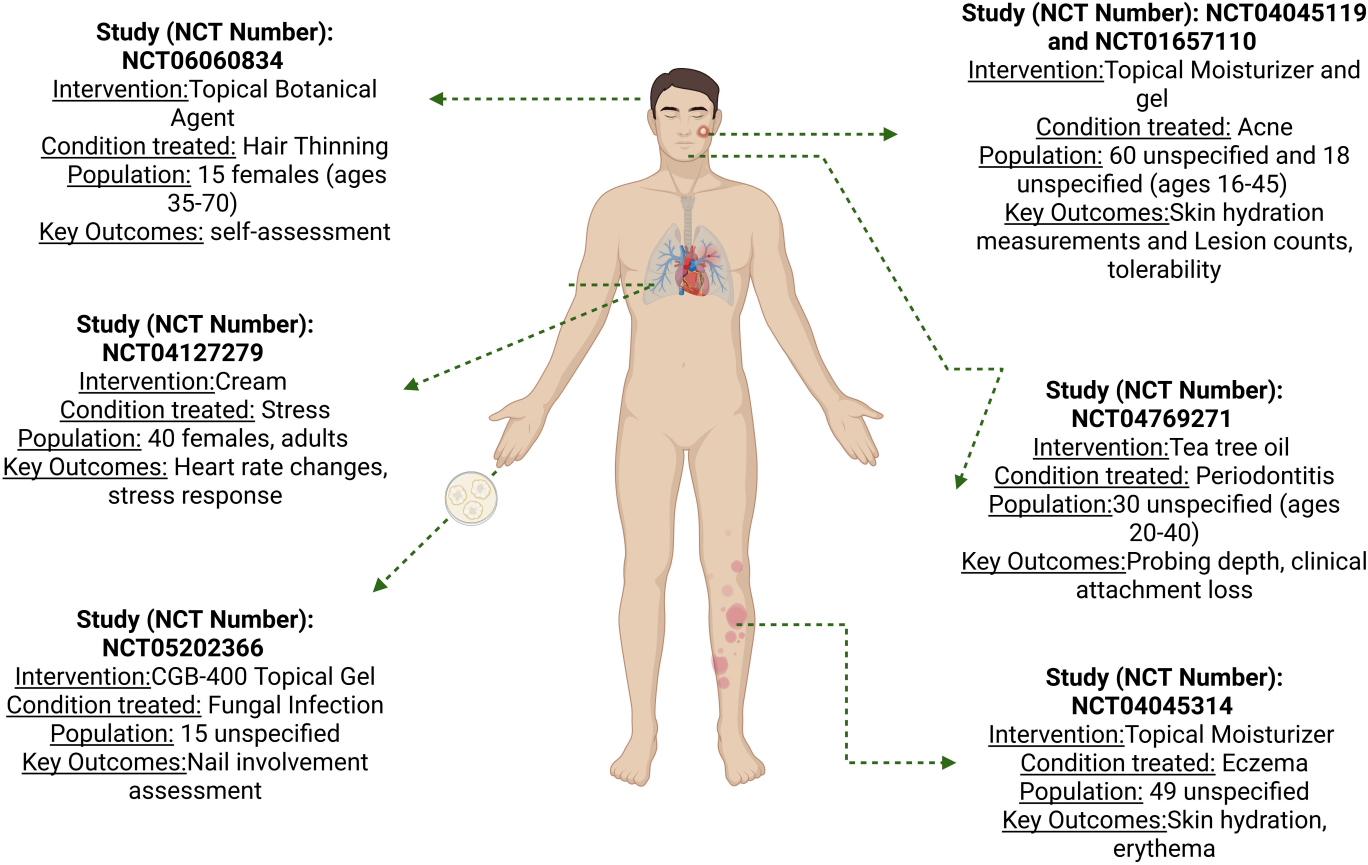
Recompilation of clinical trials of cosmetics in cosmetic applications.

Finally, these studies focus on topical products with thermal effects (heating or cooling). These trials evaluate the efficacy and safety of different gels, such as Axanova hot gel, Axanova activ gel, Perskindol Dolo Gel, Perskindol Classic Gel, Perskindol Cool Kühl-Gel, Dolor-X Hot Gel, Dolor-X Classic Gel and Dolor-X Cool Gel (whose bioactive compounds are menthol, wintergreen oil, needle oil, pine needle oil, lemon oil, orange peel oil, orange peel oil, lemon bergamot oil, rosemary oil, lavender oil, excipients) (72), whereas, this trial (73) investigated the impact of a topical nutritional product (Red Palm Oil) applied daily on women’s hair, specifically on perceived hair quality and overall well-being. It is important to note that for all the trials mentioned above, no results have yet been published on ClinicalTrials.gov for these studies (Figure 2).

A comparison of four clinical studies investigating essential oils in cosmetic formulations to treat various skin conditions is presented. The studies span different countries, including Italy, Australia, and Nigeria, with sample sizes ranging from 40 to 126 participants. The essential oils studied include argan (*Argania spinosa*), myrtle (*Myrtus communis*), oregano (*Origanum vulgare*), tea tree oil (*Tea tree oil*), and African basil (*Ocimum gratissimum*). These oils were incorporated into cosmetic formulations such as lotions, creams, and gels. Dermatological conditions treated in these studies include intertrigo, acne, and other skin conditions. The bioactive compounds present in these essential oils range from fatty acids and vitamins to specific terpenes such as eucalyptol, carvacrol, and terpinene-4-ol (Table 3).

**TABLE 3:** Clinical studies have been conducted on using essential oils in cosmetic formulations to treat dermatological conditions.

	Title article	Country of study	Population (N)	Type of essential oil	Cosmetic formulation	Biactive compound	DOI
**1**	A randomized controlled trial to compare the effectiveness and safety of adsorbent lotion containing tapioca starch, spent grain wax, Butyrospermum parkii extract, argania spinosa kernel oil, aloe barbadensis, rosehip oil, and allantoin with a low-potency topical corticosteroid in the treatment of intertrigo	unspecified	40	*Argania spinosa*	Adsorbent lotion	Tapioca Starch and Allantoin Not specified; Spent Grain Wax vegetable waxes; Butyrospermum Parkii and Argania Spinosa Fatty acids (oleic, stearic), vitamin A and E; Aloe Barbadensis Polysaccharides, vitamins (A, C, E); Rosehip Oil Essential fatty acids, antioxidants (vitamin C), and carotenoids.	doi: 10.1111/jocd.14126
**2**	Clinical evaluation of a new topical cream containing two essential oils combined with tretinoin for acne treatment	Italy	60	*Myrtus communis* y *Origanum vulgare*	Cream with essential oils and tretinoin	*Myrtus communis* eucalyptol, cineol and *Origanum vulgare* (Oregano) carvacrol	doi: 10.2147/CCID.S236956
**3**	A comparative study of tea- tree oil versus benzoylperoxide in the treatment of acne	Australia	120	Tea tree oil	Gel at 5%	Terpinen-4-ol	doi: 10.5694/j.1326-5377.1990.tb126150.x
**4**	Original Research Article Preliminary Clinical Tests on Topical Preparations of Ocimum gratissimum Linn Leaf Essential Oil for the Treatment of Acne Vulgaris	Nigeria	126	*Ocimum gratissimum*	2% oil content in alcohol (ethanol). 2% oil content in a ketomacrogol mixture base	eugenol	doi: 10.2165/00044011-200222050-00005

## 4 Discussion

Topical formulations featuring tea tree oil at 200 mg/g concentration in a gel and a facial cleanser containing 7 mg/g of tea tree oil have significantly reduced facial acne lesions (8). This underscores their potential effectiveness in managing mild to moderate acne. Furthermore, these formulations exhibited a favorable safety and tolerability profile, with only minor, transient adverse effects. Topical formulations indicate that tea tree oil-based treatments may serve as a well-tolerated alternative or complementary approach to conventional acne therapies (8). In a Phase II pilot study, 18 participants applied the products twice daily for 12 weeks. They observed a significant reduction in the total acne lesions and the investigator’s global assessment (IGA) score over time. However, no serious adverse events were reported, and minor side effects, such as flaking and dryness, were resolved without intervention. Thus, tea tree oil products significantly improve acne and are well tolerated.

It is emphasized that various studies are carried out in cell lines and *in vitro*. Such as, this study (74) investigated the concentration of tea tree oil in follicular castings after topical application of different formulations on bovine udder skin, using high-performance thin layer chromatography (HPTLC) to determine the terpinen-4-ol content, the active component of the oil, various formulations were also applied, including micro-emulsions, liposomal dispersions, multiple emulsions, and colloidal beds, finding that the microemulsion and liposomal dispersion achieved the highest concentrations in the follicles, with 0.43 and 0.41% respectively. 43 and 0.41%, respectively. These results suggest that the designed formulations may enhance the delivery of tea tree oil to the sebaceous glands, which could be beneficial for treating acne vulgaris. This is the first study (75) to quantify the research on its therapeutic efficacy. This study also investigated the effects of a cream containing 2% essential oil of *Melaleuca alternifolia* (MaEO) on wounds infected by *Taphylococcus aureus*. They evaluated its antimicrobial properties and stability in porcine and murine skin models. The results showed that the cream significantly reduced the bacterial load and improved wound healing. In addition, quality control tests were performed, confirming the formulation’s stability and safety. This approach suggests that topical application of this cream could be a promising strategy for treating infected wounds.

*In vitro* assays such as this study (76) investigated the skin penetration of tea tree oil (TTO) in its pure form and as a 20% solution in ethanol using human epidermal membranes from three donors. TTO was applied under normal conditions, and the effect of partial occlusion on the penetration of its components was evaluated. The results showed that only a tiny percentage of the TTO components penetrated the skin, with terpinen-4-ol being the primary component that managed to infiltrate. Partial occlusion increased the penetration of this compound. Similarly, this study (77) evaluated the efficacy of a topical formulation of 2% *Ocimum gratissimum* oil compared to a reference product (10% benzoyl peroxide) and a placebo in 126 patients with acne vulgaris. The formulation was found to be more effective and well-tolerated. Different formulations (single and combined extracts) were evaluated under laboratory conditions, following World Health Organization protocols. The results showed that all formulations provided good protection against mosquito bites, with the mixtures of the extracts demonstrating a synergistic effect. This article evaluates the efficacy of cosmetic formulations with essential oils for treating non-inflammatory acne in an *in vivo*, randomized, double-blind, placebo-controlled clinical study. Formulations with a blend of four essential oils (lavender, eucalyptus, mandarin, and melaleuca), pure *Melaleuca alternifolia* essential oil, a melaleuca nano-emulsion, and a placebo group were tested. The results showed that the nanoemulsion and the blend of four essential oils significantly reduced the number of comedones and improved follicular hyperkeratinization. In particular, the melaleuca nanoemulsion showed the best results, attributed to the presence of terpinen-4-ol and its ability to penetrate the skin (78). Thus, the findings suggest that formulations with essential oils may be a viable alternative in cosmetics for acne-prone skin, although it is recommended that further investigation be conducted into their long-term safety and efficacy.

Continuing with the previous line, these authors (10) evaluated the anti-aging and brightening effects of a topical serum containing 20% vitamin C, E, and raspberry leaf cell culture extract on 50 women aged 30–65. After 2 months of application to one side of the face, a significant improvement in skin color, elasticity, radiance, and smoothness was observed, as well as a reduction in scaliness and wrinkles, although some mild adverse effects, such as tingling and tightness, were reported; the results suggest that this combination of ingredients may be effective in improving the appearance of aging skin. Likewise, this study (79) investigated the extracts of the red berries of this plant as functional ingredients in cosmetics. Methanolic and aqueous extracts were obtained, and their biological activities on human fibroblasts were analyzed, as well as their ability to inhibit collagenase activity and their immunomodulatory effects on peripheral blood mononuclear cells. The results indicate that both extracts enhance fibroblast migration and have photoprotective properties. Unlike the other studies, this one evaluated (80) the oxidative stability of red raspberry seed oil (RO) under storage conditions at different temperatures (5, 25, and 40°C) for 1 month, both in pure RO and in low-energy nanoemulsions (NE). Synthetic (BHT) and natural antioxidants (ORE and OAK) were used to determine their effectiveness. The results showed that RO with BHT and ORE was stable at 5°C and 25°C, but ORE was prooxidant at 40°C. NE with biodegradable surfactants showed better physicochemical stability at room temperature and at 40°C compared to those containing polysorbate 80. This study was performed *in vitro*, evaluating the properties under controlled laboratory conditions.

Regarding sun protection, Calendula officinalis essential oil has shown potential as a UV filter. A study by Pouresmaeil evidenced that its inclusion in cosmetic formulations can contribute to skin protection against UV radiation. However, further studies are needed to determine its safety and stability in commercial products (81). This article highlights the importance of essential oils in the cosmetics and perfumery industry, emphasizing their dual role as natural sources of fragrances and as ingredients with beneficial properties for skin and hair. Among the plants mentioned, lavender is used for its soothing and healing properties, while tea tree is valued for its antiseptic and sebum-regulating action. Eucalyptus can combat photoaging caused by UV radiation, helping to reduce the appearance of wrinkles and skin dehydration (82, 83), and citrus oils, such as orange, lemon, and bergamot, are appreciated for their fresh fragrance and antiseptic action (1, 84). Finally, this article evaluates the efficacy of cosmetic formulations with essential oils for treating non-inflammatory acne in an *in vivo*, randomized, double-blind, placebo-controlled clinical study. Formulations with a blend of four essential oils (lavender, eucalyptus, mandarin, and melaleuca), pure *Melaleuca alternifolia* essential oil, a melaleuca nanoemulsion, and a placebo group were tested. The results showed that the nanoemulsion and the blend of four essential oils significantly reduced the number of comedones and improved follicular hyperkeratinization. In particular, the melaleuca nanoemulsion showed the best results, attributed to the presence of terpinen-4-ol and its ability to penetrate the skin.

## 5 Conclusion

The growing popularity of essential oils in the cosmetic industry reflects a significant shift toward more natural and sustainable products. This article highlights the efficacy of essential oils in treating various skin conditions, such as acne, atopic dermatitis, and psoriasis, thanks to their anti-inflammatory, antimicrobial, and antioxidant properties. As consumers seek alternatives to synthetic ingredients, essential oils offer multifaceted benefits that address specific skin problems and improve overall skin health.

However, regulation of their use in cosmetics remains challenging, given that prior approval is not required for commercialization. This underscores the need for more rigorous research to ensure the safety and effectiveness of these products. Formulation technologies, such as microencapsulation and nanoemulsions, are revolutionizing how essential oils are used, improving their stability and efficacy. In addition, further research into their mechanisms of action and possible interactions is crucial. Combining natural and pharmaceutical approaches can offer comprehensive skin care solutions, promoting a balance between natural beauty and modern dermatological science.

### 5.1 Limitations

Study limitations include a relatively small sample size, which may affect generalizability and the treatment duration. Several authors have stressed the need for long-term studies evaluating not only the efficacy but also the cumulative safety of daily use of essential oils, as chronic exposure could induce skin sensitization or toxicity in specific individuals. The absence of standardized protocols for evaluating irritation, allergy, and phototoxicity represents a significant gap in current literature.

Likewise, no post-treatment follow-ups were performed to observe the durability of the results. It is also essential to consider that the studies focus on different genders and age ranges, which may not reflect the effectiveness of the treatment in other populations. Finally, self-assessment of satisfaction and adverse reactions may be subject to reporting biases.

In addition to the previously mentioned methodological and regulatory limitations, other studies have highlighted additional constraints that affect the robustness and applicability of the results on the use of essential oils in dermo-cosmetic products. One of the main limitations is the high variability in the chemical composition of essential oils, even when they come from the same plant species. Factors such as geographical origin, cultivation conditions, extraction method, and storage can significantly alter the concentration and profile of bioactive compounds, making the reproducibility of the effects observed in different investigations difficult.

Another significant limitation is the paucity of studies evaluating the impact of essential oils in diverse populations, including different age groups, skin types, and specific dermatological conditions. Most clinical trials have been conducted on young adults and those with mild conditions, so the same efficacy and safety cannot be assured in children, older adults, or people with chronic skin diseases. Also, although nanoencapsulation technologies and lipid systems have been shown to improve the stability and bioavailability of essential oils, there are few direct comparative studies between these advanced formulations and conventional ones. The lack of data on pharmacokinetics, skin metabolism, and interaction of essential oils with other cosmetic ingredients limits the comprehensive understanding of their behavior in the skin.

The results obtained in this research on using essential oils in cosmetic products have important implications for practice, policy, and future research. In clinical practice, health professionals could consider essential oil-based products as adjunctive treatments for common skin conditions such as acne, atopic dermatitis, and psoriasis, given their anti-inflammatory and antimicrobial efficacy. The need for stricter regulations is highlighted in terms of policy, as the lack of prior approval by agencies such as the FDA puts consumer safety at risk, warranting a more thorough review of current standards. For future research, larger-scale and more extended clinical studies that evaluate the efficacy of these products and their mechanisms of action and long-term safety are essential. In addition, integrating new formulation technologies, such as microencapsulation and nanoemulsions, represents a promising avenue to optimize the stability and bioavailability of active compounds in essential oils.

## Data Availability

The original contributions presented in this study are included in this article/supplementary material, further inquiries can be directed to the corresponding author.
